# Oromandibular Limb Hypogenesis Syndrome Type IIB: Case Report of Hypoglossia-Hypodactyly

**DOI:** 10.1155/2013/370695

**Published:** 2013-02-04

**Authors:** Manasa Anand Meundi, Gopakumar R. Nair, Prathima Sreenivasan, A. C. Raj

**Affiliations:** ^1^Department of Oral Medicine, Diagnosis and Radiology, Dayananda Sagar College of Dental Sciences, Shavige Malleshwara Hills, Kumaraswamy Layout, Bangalore 560078, India; ^2^Department of Oral Medicine, Diagnosis and Radiology, New Horizon Dental College & Research institute, Sakri, Bilaspur 495001, India; ^3^Department of Oral Medicine, Diagnosis and Radiology, Kannur Dental College, Anjarakkandy post, Kannur 670612, India; ^4^Department of Oral Medicine, Diagnosis and Radiology, KVG Dental College and Hospital, Vidhyanagar post, Kurunjibagh, Sullia 574327, India

## Abstract

Hypoglossia-hypodactyly is a rare congenital anomaly affecting the tongue and the limbs. Hall in 1971 classified it under a complex group of disorders called oromandibular limb hypogenesis syndromes. It is an extremely rare condition with around 40 cases reported in the world literature. The cause of the syndrome is unknown. Some type of intrauterine trauma is the most widely accepted etiology. The characteristic features of the syndrome are hypoglossia, limb anomalies of variable degree, and micrognathia of the mandible. This unique case report of hypoglossia-hypodactyly was observed in a patient with normal mandible. In addition, patient also had pulmonary regurgitation. His parents and other siblings were normal. Positive prenatal history of maternal hyperthermia was obtained suspecting it to be the cause of the syndrome.

## 1. Introduction

Hypoglossia is a rare congenital anomaly [1]. It was first described by de Jussieu in 1718 as Aglossia. It may occur in isolation or in conjunction with anomalies of the extremities. Rosenthal in 1932 reported the first case of such an association as “aglossia-adactylia” [2]. Subsequently, similar cases were documented but with variable terminologies like aglossia-congenita, partial adactylia, aglossia-adactylia syndrome, partial anodontia, and microglossia [3]. In 1971, Hall BD coined the term “hypoglossia-hypodactylia syndrome,” as he observed that the tongue and limbs were never totally absent. He considered it as a part of classification of oromandibular limb hypogenesis syndromes (OLHS) [4]. The prevalence of hypoglossia-hypodactyly has been documented as less than one per million population [5]. Around 47 cases have been reported so far [1].

The etiology of hypoglossia-hypodactylia (HG-HD) is unknown. Both genetic and environmental factors have been proposed to be responsible for the occurrence [6]. Most cases occur sporadically to unrelated parents with single affectation in the family [3]. Mishima et al. suggest an autosomal dominant or multifactorial inheritance with reduced penetrance and variable expression [1]. Mutation of *Msx 2*, a homeobox gene located in the mesenchyme of branchial arches and limb buds, as well as in the developing teeth and the alveolar ridges, may also be responsible for the clinical manifestations [7].

Environmental factors likely to be the etiology are maternal exposure to radiation and teratogenic drugs, intrauterine trauma/vascular accidents, chorionic villous sampling procedures, and maternal hyperthermia. Membranous strands resulting from amniotic rupture or chorionic villous sampling procedures during early pregnancy may interfere with oral and limb development [3, 8]. Reports of exposure to drugs like Tigan, Benedictine, Imipramine, Diazepam, Chlorpromazine, and Meclizine [2, 3] suggest their involvement, but their effect in the causation of this syndrome has not been proved. Fetal exposure to radiation during first trimester (a crucial period for orofacial development) may result in anomalies at birth [9]. Premature involution or injury to the stapedial artery supplying the first branchial arch may interfere with tongue vasculature [3]. Similarly, intrauterine vascular occlusion from placental fetal vessel thrombosis or emboli, followed by aseptic tissue necrosis, may cause peripheral amputation of the limbs [3]. Hyperthermia is a proven teratogen both in animals and humans. Maternal hyperthermia during first trimester of pregnancy can result even in fetal death [10]. According to Gorlin, intrauterine trauma [6] is the most widely accepted cause of this syndrome.

The three features essential for the diagnosis of this syndrome are [3]

1. variable reduction in the tongue size (microglossia);
2. micrognathia of the mandible (or maxilla) in the midline segment;
3. limb anomalies of varying severity.

Size of the tongue varies from total absence (aglossia) to negligible hypoglossia. It is associated with a small mandible, a receded chin, atrophic mandibular anterior alveolar ridge, and missing incisors. Other oral and extraoral changes observed are enlarged sublingual ridges, defective lower lip, hypertrophic major salivary glands, mandibular cleft in the midline, fibrotic bands connecting the lower lip to the alveolar ridge, and gingival abnormalities [6].

The limb anomalies are extremely variable ranging from syndactyly (incomplete separation of the fingers) to amelia (missing limb) [3, 6]. In general, they tend to be distal to humerus and femur [2]. One or more limbs may be affected with variations seen in the same individual as well as in different patients [2, 3, 6].

Visceral anomalies associated with this syndrome include fused labia majora, imperforate anus, absence of a kidney, and ileal atresias [3].

HG-HD is an extremely rare condition with as few as 47 cases being reported in the world literature [1]. Among them, only three syndromic patients presented with a normal mandible [3, 11]. Yet, they had deficient premaxilla. This paper aims to report an unusual case of hypoglossia-hypodactylia without micrognathia of the mandible or maxilla, who in addition also had pulmonary regurgitation.

## 2. Case Report

A twenty-two-year-old male with extensive limb deformities reported to the department of Oral Medicine, Diagnosis, and Radiology with a chief complaint of unclear speech and difficulty in tongue movements.

The patient's medical history was insignificant. He was the fourth child of normal and unrelated parents. He had four siblings: one brother and three sisters all of whom were normal.

His prenatal history revealed that his mother had four episodes of fever during her first trimester and was medicated for the same. She was unable to remember the names of the medications. She had spontaneous vaginal delivery after thirty-nine weeks of gestation to give birth to a male baby with multiple limb deformities. Patient's family history for congenital abnormalities was negative.

**Figure 1:**
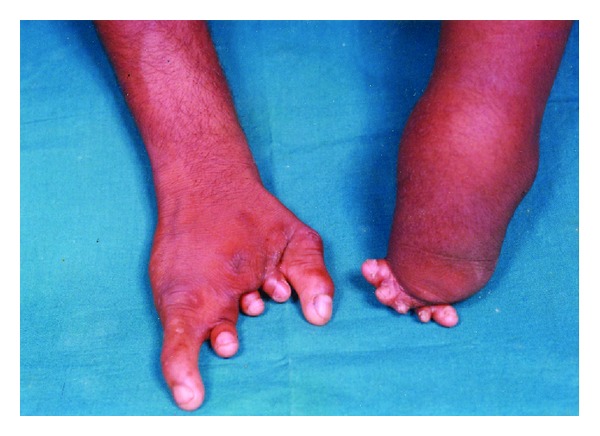
Showing both the upper limbs of the patient. Right limb demonstrates hypoplastic fingers (brachydactyly). The left forearm is absent with five nubbins attached to its distal part.

**Figure 2:**
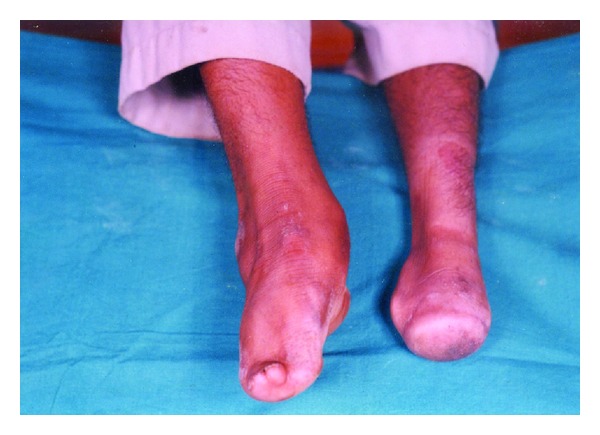
Showing both the lower limbs of the patient; Right foot is hypoplastic, and left foot is absent (apodia) with all the ten toes missing.

**Figure 3:**
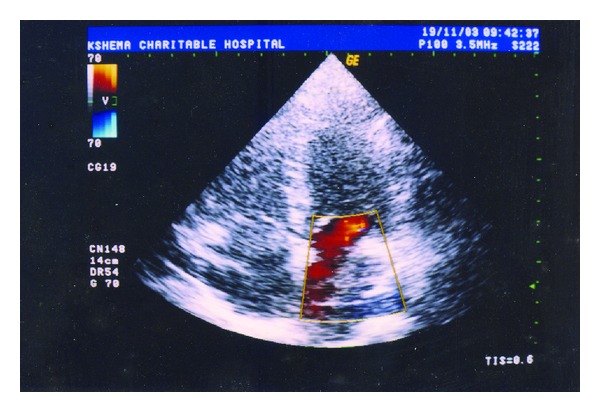
Color Doppler ultrasonographic image showing the regurgitation of blood into the left atrium.

On general examination, a healthy, moderately built male of normal intelligence presented to the clinic with an altered gait. He had anomalies affecting all the four limbs. His right hand showed brachydactyly (short digits) of second to fourth fingers with hypoplastic thumb and little fingers. Finger nails appeared normal (Figure 1). In the left hand, hemimelia (missing half the limb) was observed below the elbow with five nubbins attached to its distal part. Radiograph of the right hand showed hypoplastic second and third metacarpals with deficient second to fourth phalanges. Absence of distal part of the radius and ulna was noted in the left hand radiograph with subluxation of the superior radioulnar joint. Lower limbs were also severely deformed. Right limb showed hypoplastic foot with adactyly (missing digits). Left limb had apodia (missing foot) with adactyly (Figure 2). Radiographically, right limb revealed hypoplasia of the phalanges and first metatarsal bone; left limb revealed absence of tibia, fibula, and both the malleoli. Detailed systemic clinical examinations with appropriate investigations were also performed. Though patient was clinically normal, his chest radiograph showed prominent pulmonary bay. Further, Color Doppler ultrasonography revealed pulmonary artery dilatation with pulmonary regurgitation (Figure 3).

**Figure 4:**
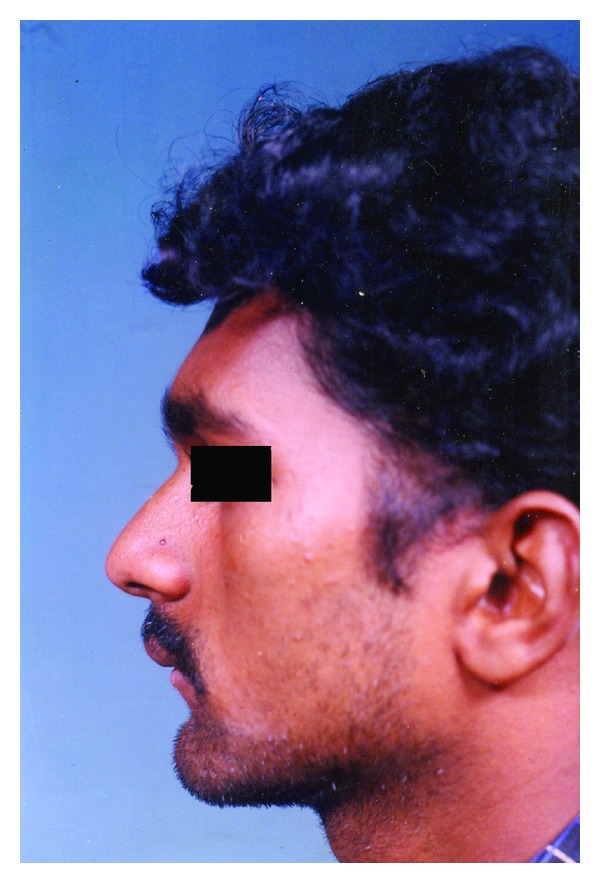
Left lateral view of the patient showing convex profile, competent lips, and deep mentolabial sulcus.

On extraoral examination, his lips were competent; profile was convex with a deep mentolabial sulcus (Figure 4).

**Figure 5:**
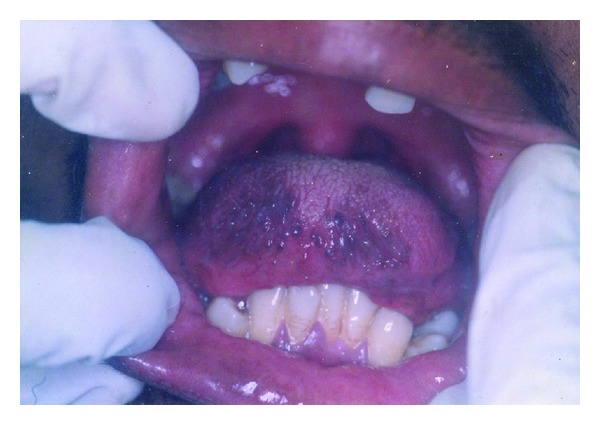
Intraoral view of the small tongue showing a poorly defined tip.

**Figure 6:**
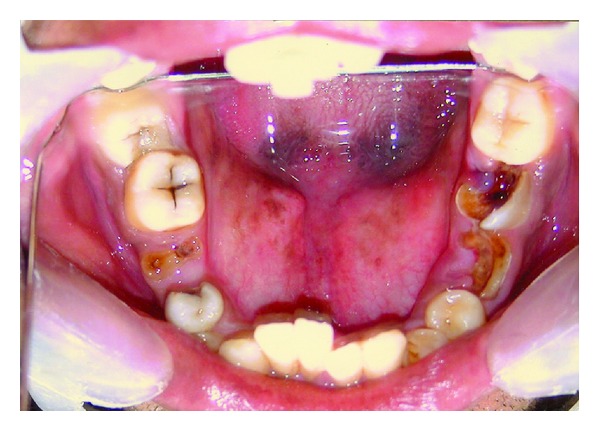
Intraoral view of the mandibular arch demonstrates the lingual frenum attaching the blunt tongue tip to the floor of the mouth far behind the incisors. Root stumps of molars are evident. Missing right central incisor may be noted.

Intraorally, his tongue was hypoplastic; about two thirds the normal size with a rounded tip (Figure 5). The tip was attached to the floor of the mouth by a short lingual frenum and positioned about a centimeter behind the lower incisors (Figure 6). This restricted the protrusive and lateral movements of the tongue. But speech was affected minimally.

**Figure 7:**
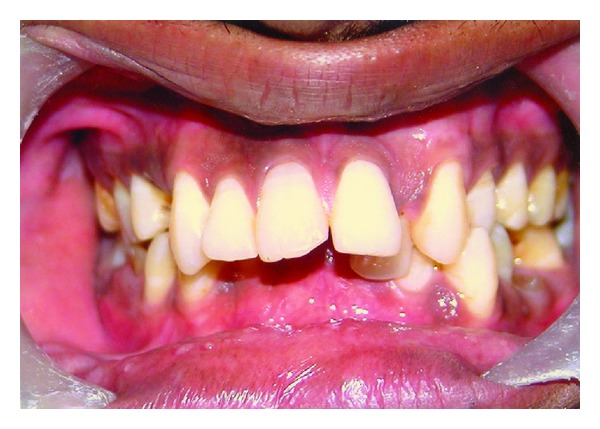
Intraoral view of the teeth in occlusion showing anterior deep bite and palatally placed maxillary left lateral incisor.

Mandibular anteriors were retroclined with congenitally missing right central incisor (Figure 6). There was no radiographic evidence of impaction. Palate was V shaped, high arched, and narrow. Maxillary left lateral incisor was palatally positioned, thus blocking the regular alignment of mandibular anteriors. Anterior deep bite was observed (Figure 7). Root stumps of maxillary left first premolar, mandibular first molars, and mandibular right second molar were present (Figure 6).

Presence of a small tongue in association with extensive limb deformities led to the diagnosis of oromandibular limb hypogenesis syndrome type IIB, hypoglossia-hypodactyly. Although patient complained of altered speech and difficulty in tongue movements, he refused to undergo surgical procedure for the correction of the same. Basic oral rehabilitation procedures like extraction of the root stumps and oral prophylaxis were performed.

## 3. Discussion

OLHS (OMIM 103300) [5] represents a spectrum of disorders affecting the tongue and the limbs. Such patients often present with overlapping clinical features. Hence, in order to simplify the diagnosis, this complex syndrome has been classified twice. Hall's classification [6] is ideal to define the limb anomalies expressed in this patient. Accordingly, this case is OLHS type IIC: hypoglossia-hypodactylomelia, thus expressing the left hemimelia in the diagnosis. Since hypoglossia was the major determinate for the categorization, Chicarilli and Polayes [4] proposed a classification considering both embryologic origin and clinical features. He recognized four major classes, wherein type II represents microglossia as the primary disorder. Accordingly, this case is OLHS type IIB: hypoglossia-hypodactyly.

HG-HD is etiologically heterogeneous. The present case gave a positive prenatal history of maternal fever. Evidence of maternal hyperthermia causing OHLS exists [10, 12]. Maternal fever at/above 102°F between 4–14 weeks of pregnancy results in a range of defects including limb reduction, central nervous system (CNS) defects, facial dysmorphogenesis, and fetal death. Heat induced vascular disruption of the embryo has been implicated in the pathogenesis, The nature of anomalies is related to the extent, duration, and timing of the maternal fever. CNS defects appear to be the most common consequence of gestational hyperthermia. Yet, patients with HG-HD are often born with normal intelligence [10]. Based on this, there is a need to explore maternal hyperthermia as a cause of the syndrome.

Hypoglossia, limb anomalies, and micrognathia are the three characteristic features of the syndrome [3]. Of the three, latter two are often considered diagnostic because assessment of hypoglossia is subjected to observer variability (especially if it is minimal) because tongue is a muscular organ and need to be examined both at rest and during function [3]. The extent of limb deformities varies from syndactyly (incomplete separation of the fingers) to amelia (complete loss of the limb). Severe micrognathia is observed in all the cases of OLHS, due to the osseous defect in the mandibular midline region, and occasionally involves the premaxilla. But this patient of HG-HD presented with a normal mandible. Two similar cases were reported by Lustmann et al. [3], but his patients had a deficient premaxilla instead of mandible. This patient also suffered from pulmonary regurgitation, which probably has never been reported to be associated with this syndrome.

HG-HD syndrome is diagnosed at birth. Though tongue is deficient, in majority of cases, activities like swallowing and speech improve with time. Other muscles substitute for tongue and assist in feeding. Speech therapy improves phonetics. Surgery is desirable only if hypoplasia is severe. Prosthetic limbs would improve the locomotion and enhance the quality of life.
